# What Does the Pharmaceutical Industry Look for in Pharmacology Graduates? A Thematic Analysis of Semi‐Structured Interviews With Pharmaceutical Industry Employees on Their Perceptions of Pharmacology Graduates

**DOI:** 10.1002/prp2.70335

**Published:** 2026-10-21

**Authors:** John Broad

**Affiliations:** ^1^ Department of Pharmacology, Faculty of Life Sciences and Medicine King's College London London UK

## Abstract

The pharmaceutical industry is a major employer of graduates from pharmacology BSc and MSc programs. Understanding its expectations of graduates is therefore important for pharmacology educators. To explore this, interviews were conducted with pharmaceutical industry employees to understand their views on graduate colleagues. Eight semi‐structured interviews were conducted with employees of the UK pharmaceutical sector, identified by convenience sampling. The transcripts of the interviews were coded, and the resulting 117 codes were used to generate three themes and nine sub‐themes. The first theme concerned how academia should prepare students for industry careers. The subthemes included what academia should focus on in their courses; differences identified by interviewees between industry and academia; educating students about the reality of careers in industry; and highlighting career options outside of academia via industrial placements. The second theme concerned the graduate attributes desired by industry, with the subthemes of knowledge, skills, behaviors and attitudes. Finally, the third theme concerned the behaviors and attributes students should develop to be successful in industry. These findings provide insight into graduate preparation for the workplace and may inform pharmacology program design. Exploring the views of employees in other career destinations of pharmacology graduates would be valuable. Limitations include convenience sampling, the limited number of participants, and the potential for interviewer bias.

## 1 Introduction

The pharmaceutical industry has historically been a major employer of graduates from undergraduate and postgraduate pharmacology courses [1]. Understanding its requirements is important for educators preparing students for their career after graduation. Both current pharmacology students and pharmacology graduates have challenges in identifying employment opportunities and pursuing career goals, a problem which is more challenging for graduates than for students [2]. Graduates observe a disconnect between university education and workplace expectations [3].

Pharmacology education has moved away from the didactic transfer of knowledge to incorporate a wide range of active learning techniques [4], such as flipped classrooms, case‐based, team‐based, and problem‐based learning. Despite the widespread use and implementation of these innovative pedagogical approaches, a disconnect between university education and workplace expectations remains. These approaches do not necessarily guarantee students will develop appropriate employability skills. Pharmacology educators should seek to align the learning outcomes of their courses with the aspirations of their students, and future curriculum revisions may incorporate a greater emphasis on the development of career‐relevant skills. One way of ensuring pharmacology educators can create curricula that develop the appropriate knowledge, skills, and attributes required by industry is to ask current colleagues of pharmacology graduates what they value in graduates.

Semi‐structured interviews with employers of pharmacology graduates allow a valuable perspective to be uncovered. In‐depth interviews allow the exploration of individual experiences and provide a rich understanding of industry employees' perceptions of graduates [5]. Using external stakeholders to inform curriculum revisions can lead to a course more closely aligned with societal needs [6]. The aim of this study is to uncover pharmacology industry employees' opinions on current graduates, and to understand the personal and professional qualities required for students to succeed in this industry.

## 2 Methodology

Ethical approval for this study was granted by the King's College London local ethics committee, ethical approval reference number LRS/DP‐24/25‐45540.

### 2.1 Participant Recruitment

The interviewees were identified by convenience sampling and were recruited through a variety of ways. Primarily, participants in the personal network of the research team were contacted. Other participants were invited to join the study following discussions at conferences, or through wider personal connections.

### 2.2 Participant Information

Eight employees of the UK pharmaceutical sector agreed to be interviewed. The interviewees are currently employed by small, medium and large pharmaceutical companies, a contract research organization, and one interviewee was an independent contractor who has experience of working for a large pharmaceutical company. Pre‐clinical and clinical pharmacology areas were represented. Participants had a median of 12.5 (4–26) years' experience in the pharmaceutical industry. Two participants (IEvv and v4rC) reported that they had worked with students from King's College London (Figure S1). Participants completed consent forms prior to taking part in the study, and were given the opportunity to withdraw from the study for at least 1 month from the date of the final interview. No participants withdrew from the study following the interview.

### 2.3 Interview Process

Following informed consent, seven interviews were conducted on MS Teams, and one interview was conducted in person. The interviews were based on the interview questions found in Figure S1. All interviews were recorded on MS Teams and transcripts were automatically generated. These transcripts were reviewed and amended for accuracy and approved by the interviewee prior to analysis. Participants were also pseudonymized at this stage by the interviewer by using a random alphanumeric generator. The transcripts were then coded using NVivo 15. The codes were reviewed and organized into nine subthemes using mind maps, and again into three themes [7] (see Figure 1). 7/8 participants gave their permission for the redacted transcripts to be shared via the King's Open Research Data System https://figshare.com/s/44550ebf5bc8e8c4c141.

**FIGURE 1:**
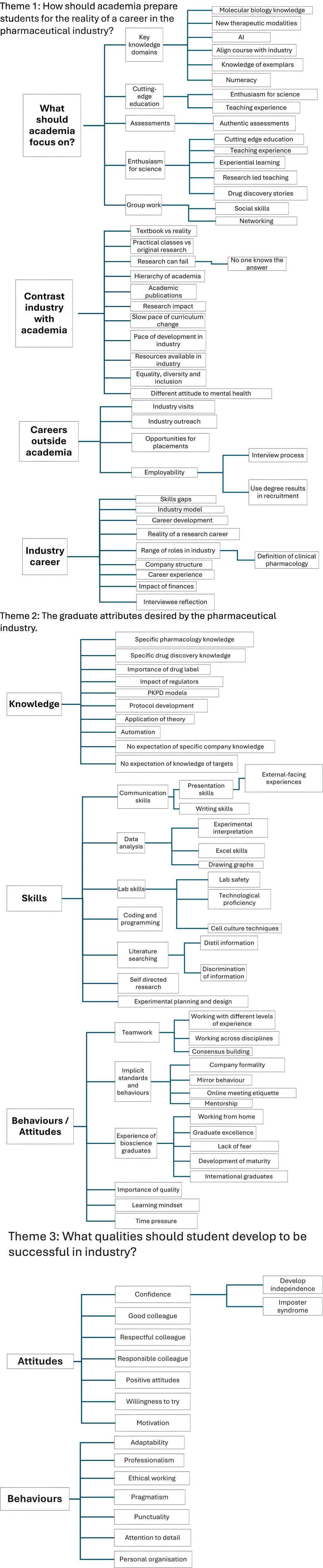
Mind maps constructed from the 117 codes. The codes were organized into subthemes using an iterative mind mapping approach. The subthemes are found on the left of the image, with direct linkages to codes, and with related subcodes linked indirectly to these subthemes where appropriate.

## 3 Results

The interviews lasted between 22 and 58 min. In summary, over the eight interviews, 117 codes were generated. There were three main themes generated from these codes.

### 3.1 Theme One: How Should Academia Prepare Students for the Reality of a Career in the Pharmaceutical Industry?

Theme one was developed from four subthemes. The first subtheme discussed what academia should focus on when preparing students for their pharmaceutical industry careers. These included giving students knowledge of exemplars of drug discovery targets or pathways, developing group work skills and nurturing students' enthusiasm for research.

Knowledge of exemplars was seen to be a practical way of addressing the gap between the wide range of targets industry could be working on, and what students were expected to know on coming into industry (mentioned by four interviewees). Group work was identified as important by five interviewees. Participant *sDBu* recognized that teamwork is now more emphasized in university education compared to their own experience (Table S1A, Subtheme 1). Interviewees suggested that inculcating an enthusiasm for biomedical research was something that academics currently did well and should continue to nurture students' passion for research (five interviewees; Table S1A, Subtheme 1).

The second subtheme concerned the contrast between industry and academia including the differences between practical classes and research. Participants identified that practical classes, which are designed to work every time, and the outcome is already known, provide insufficient preparation for the uncertainty inherent in working in a “real” laboratory (five interviewees). Awareness of the slow pace of curriculum change in academia was compared to the rapid pace of change in industry by participant *12ep*. Some participants explicitly contrasted the objectives, professionalism, pragmatism, and individuality of academic and industry research (six interviewees; Table S1A, Subtheme 2).

The third subtheme concerned more general discussions of careers outside of academia. Participants often stated that they were unsure about industry during their own time at university. Sandwich years or similar were highlighted as an effective way to increase students' awareness of the pharmaceutical industry (five interviewees; Table S1A, Subtheme 3).

The fourth subtheme concerned the role academia has in educating students about the reality of careers in industry. Site visits by students and industry employees visiting university campuses were both suggested ways of increasing exposure of students to the industry (two interviewees; Table S1A, Subtheme 4).

### 3.2 Theme Two: The Graduate Attributes Desired by Industry

Theme two arose from the subthemes of knowledge, skills, behaviors and attitudes desired by industry. Specific knowledge of pharmacology and drug discovery was seen as important for graduates by all eight participants. Some participants suggested that many graduates employed by the pharmaceutical industry have good biomedical science knowledge but lack some basic understanding of pharmacology (four interviewees; Table S1B, Subtheme 1).

The skills required by industry were highlighted by interviewees. Skills commonly mentioned included data analysis skills and numeracy (six participants), communication skills including presentations (seven participants), and laboratory skills (seven participants; Table S1B, Subtheme 2).

The ability to work across disciplines, collaborate with colleagues at different levels of experience, and build consensus was valued (five interviewees). In general (and in contrast to some media perceptions) graduates were perceived to be excellent colleagues (three interviewees). Implicit industry workplace standards and expectations of formality were highlighted and contrasted with academia (four interviewees; Table S1B, Subtheme 3).

### 3.3 Theme Three: What Qualities Should Students Develop to Be Successful in Industry

Subtheme one arose from codes suggesting that behaviors such as professionalism (five interviewees) and an attention to detail were valued (two interviewees). Some discussed the different expectations between university and the workplace and participant *12ep* reflected on their own challenges in managing this transition (Table S1C, Subtheme 1).

Subtheme two concerned the attributes students should develop to be successful in industry. A positive attitude (four participants), a willingness to try new tasks (one participant), and the ability to be a respectful and responsible colleague (one participant) were all valued. In the main, graduates displayed these features, and enthusiasm and the positivity of graduates were particularly valued by four interviewees (Table S1C, Subtheme 2).

## 4 Discussion

This study elicited opinions from pharmaceutical industry employees regarding their perceptions of recent pharmacology graduates. The generalizability of this exploratory, qualitative study is limited by the relative homogeneity of the small sample population, and the unblinded convenience sampling methodology used is prone to bias. Despite this, highly positive perceptions of graduates, alongside areas of current challenge, were found. Overall, industry employees regarded graduates highly, noting their positive behaviors and attitudes in the workplace. The enthusiasm graduates display for biomedical science and drug discovery, embedded (at least in part) in students by university educators, was recognized and appreciated by the interviewees.

Industry employees recognized the importance of specific pharmacological knowledge. For some, the utility of pharmacology in drug discovery is implicit, but knowledge of the foundational principles of pharmacology, and how pharmacology as a discipline underpins drug discovery, was absent in some graduates. Interviewees did not expect graduates to be aware of every potential target they were or could be working on, but having knowledge of at least one example of a receptor, enzyme, or signaling pathway that can be modified for therapeutic benefit was seen to be important. The IUPHAR core concepts of pharmacology could be used by educators to help distill which core pharmacology knowledge should be included in their curriculum [8].

Similarly, the appreciation of the value of the development of laboratory skills was made, emphasizing the importance of supporting the provision of these opportunities for students. Innovative approaches to continue to supply practical laboratory experiences to students may be required to support the development of these skills. Most participants also recognized the importance of data analysis skills and numeracy in the workplace, a finding supported by other studies [9]. Focused support for the students that struggle with numeracy could help address this skill deficit.

Importantly, the skills valued by industry employees are not always popular with students and academics. Students report experiencing “stage fright” as a significant obstacle to delivering presentations [10]. For this (and other) reasons, students and educators may avoid using presentations as an assessment modality. This may inadvertently have a detrimental effect on the development of skills particularly valued by employees, in turn having an effect on student employability.

Group work can be seen to be a challenge for students, administrators and academics, with free‐riding having a detrimental effect on student satisfaction [11]. Industry works in groups, but academic credit is issued individually. Again, the data here suggest these forms of activities should be maintained for their beneficial impact on graduate employability.

Providing placement opportunities in industry was seen by most interviewees as an important way of demonstrating the reality of industry careers to students. Undergraduate practical sessions were seen to be inadequate preparation for the reality of working in a “real” laboratory, despite the acknowledgement that they are useful in developing other practical skills. Exposure to industry by site visits, industry partners delivering talks or even academic content could be ways to address the perceived lack of understanding of industry in students.

Finally, supporting students in their development of their sense of professionalism and collegiality will prepare them for roles in the pharmaceutical industry. These behaviors are not typically captured by traditional courses and assessment methods. A way to address this may be to include well‐designed reflective, authentic and/or problem‐based assessments in the course design to help students prepare for the workplace [12].

The domains highlighted in this study are similar to those identified as important in employers of postgraduate students [13]. Breadth and depth of knowledge, communication skills, data analysis skills, experiential training, and adaptability were highlighted by both studies. This overlap suggests that despite differences in educational attainment, undergraduate and postgraduate students require similar attributes to thrive in the pharmaceutical industry.

Some important knowledge and skill domains were not raised by the study participants, including six of the seven top priority areas highlighted by the recent Association of the British Pharmaceutical Industry report [14], with pharmacokinetic/pharmacodynamic modeling being the exception. In addition, language and linguistic skills may have been raised if this study included participants from outside of the UK. This study provides a basis for a larger survey incorporating the perceptions of employees across the range of careers undertaken by pharmacology graduates. This will help inform future curriculum revisions of pharmacology courses.

## 5 Limitations and Reflexivity

A major limitation of this study is that only participants employed in the UK pharmaceutical industry were interviewed, limiting the generalizability of the findings. Many pharmacology graduates undertake careers outside of industry. Participants were recruited through convenience sampling and may therefore not be representative of all industry viewpoints, and the views of a limited number of participants were included in the study. The codes were generated by one researcher (a former industry employee, and current pharmacology educator) with a deep understanding of the interviews, contributing to the potential for bias. Therefore, amendments to undergraduate curricula should not be made solely on the findings of this study.

## Author Contributions

**John Broad:** conceptualization, investigation, writing – original draft, writing – review and editing, project administration, formal analysis.

## Conflicts of Interest

The author declares no conflicts of interest.

## Supporting information


**Figure S1:** Semi‐structured interview topic guide.

**Table S1:** Representative quotes from participants that were coded prior to organization into subthemes and themes. The participants pseudonymized identifiers are found in brackets after the quote.

## Data Availability

The transcripts from 7/8 participants that support the findings of this study are openly available in King's Open Research Data System at https://figshare.com/s/44550ebf5bc8e8c4c141. The other transcript is available on request from the corresponding author due to privacy restrictions.
