# Supplementary material for: What Does the Pharmaceutical Industry Look for in Pharmacology Graduates? A Thematic Analysis of Semi‐Structured Interviews With Pharmaceutical Industry Employees on Their Perceptions of Pharmacology Graduates
Source: Pharmacol Res Perspect. 2026 Oct 21;14(6):e70335. doi: 10.1002/prp2.70335 (PMC13685062; doi:10.1002/prp2.70335)
Supplement: Supplementary file 1 [file PRP2-14-e70335-s001.docx]

**Supplementary figure 1: Semi-structured interview topic guide**

What experience have you got in employing bioscience graduates? Are any of those graduates from either the pharmacology course or other biomedical science course at King’s College London?

What knowledge do you think students need to have when they start working with you? Do you think they generally possess this knowledge?

What skills would you expect a pharmacology or bioscience student to possess on day 1 of their employment with you? Are they able to do these things, in your experience?

How do you expect students to approach their work, and behave in your institution? Do graduates behave in this way? Do they demonstrate the attitudes you have mentioned?

What do you know about how students are assessed at university? Are the assessments you know about related to what students should be able to do in your workplace? What would you like to see students assessed on?

In your opinion, in our role as university biomedical science teachers preparing students for their career, what do we do well? What gaps do you think exist?

Summing up: Overall, if you were in my position, what would you focus on to prepare student better for the workplace?
